# Coronary Artery Disease Screening With Carotid Ultrasound Examination by a Primary Care Physician

**DOI:** 10.14740/cr456w

**Published:** 2016-02-20

**Authors:** Arihide Okahara, Kenji Sadamatsu, Taku Matsuura, Yasuaki Koga, Daigo Mine, Keiki Yoshida

**Affiliations:** a Department of Cardiology, Saga-Ken Medical Centre Koseikan, Saga, Japan

**Keywords:** Computed tomography, Coronary angiography, Coronary risk factor, Asymptomatic, Coronary intervention, Intima-media thickness

## Abstract

**Background:**

In this study, we investigated the feasibility of primary care physicians using carotid ultrasound to perform coronary artery disease screening in asymptomatic patients with multiple coronary risk factors.

**Methods:**

We retrospectively collected the data of 135 consecutive asymptomatic patients (mean age: 68.5 ± 8.4 years; male, 75%) who were referred to our institution due to abnormal findings on a carotid ultrasound performed by a primary care physician and who underwent coronary computed tomography angiography.

**Results:**

The mean number of risk factors was 4.1 ± 1.2 and the mean intima-media thickness was 2.00 ± 0.63 mm. Mild (≤ 50%), moderate (51-75%), and severe (> 76%) coronary stenosis was observed in 54 (40%), 27 (20%), and 25 patients (19%), respectively, while no plaque was found in 24 patients (18%), and five patients (4%) could not be evaluated due to calcification. Consequently, coronary angiography was performed in 56 (41%) patients and coronary intervention was required in 31 patients (23%). A multivariate logistic regression analysis demonstrated that the ratio of low-density lipoprotein cholesterol levels to high-density lipoprotein cholesterol levels, the use of calcium channel blockers and the value of the diastolic blood pressure were related to > 50% coronary stenosis.

**Conclusions:**

The use of carotid ultrasound in the coronary artery disease screening by primary care physicians resulted in a high prevalence of coronary artery disease and high probabilities of coronary angiography and revascularization, and thus it is considered to be a useful and feasible strategy for the screening of asymptomatic patients.

## Introduction

Global deaths from ischemic heart disease increased by 41.7% between 1990 and 2013, despite a decrease in age-specific death rates; the increase is attributed to the aging of populations and population growth [1]. Although the prevention of ischemic heart events has become an important goal, fatal coronary disease is the first manifestation of ischemic heart disease in a large proportion of asymptomatic patients [2]. An effective strategy is therefore needed to screen high-risk asymptomatic patients for coronary artery disease. At present, no such screening strategies have been established [3].

Recently, a number of studies have shown a close association between carotid atherosclerosis and coronary artery disease [4-10]. In addition, several studies from one university hospital have reported the usefulness of carotid ultrasound in the screening of diabetic patients for coronary artery disease [11-13]; however, it is generally difficult and time-consuming to perform precise measurements on carotid ultrasound, and sonography training and strict adherence to quality control are necessary [14]. Therefore, it remains unclear whether screening with the use of carotid ultrasound can be applied to daily practice in the real world.

We have encouraged primary care physicians in our area to conduct carotid ultrasound examinations in patients with multiple coronary risk factors since 2008. We herein investigate the usefulness and feasibility of the implementation of carotid ultrasound examinations in screening for coronary artery disease at the clinics of primary care physicians.

## Methods

### Screening strategy and study population

**Figure 1:**
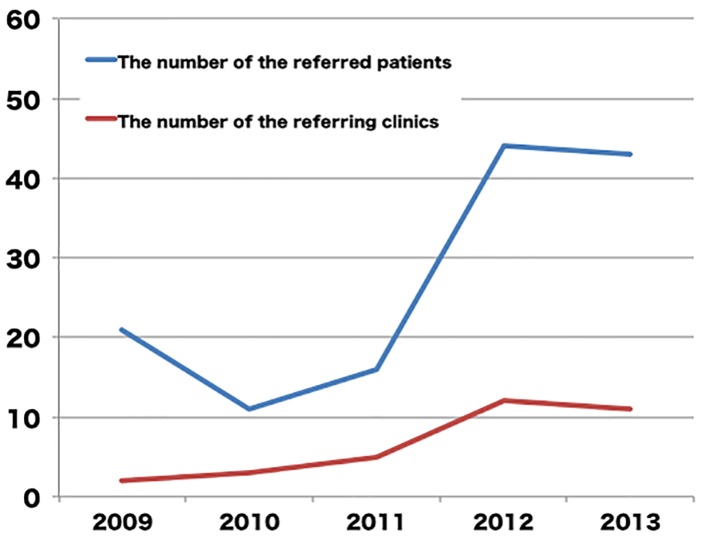
The annual changes in the number of the patients referred to our hospital and in the number of clinics who referred patients to our hospital for further evaluation of coronary artery disease.

The study was conducted in Saga central medical district, which is located in the western part of Japan. This district covers an area of 793.15 km^2^, and has a population of 353,347; 23.4% of the population is over 65 years of age [15]. The district has 158 internal medicine clinics and two major medical centers with cardiac catheterization laboratories, including our own. We have encouraged the primary care physicians in our area to conduct carotid ultrasound in patients with multiple coronary risk factors for the screening of coronary artery disease since 2008. Coronary risk factors were defined as male gender, age older than 45 years in men and 55 years in women, diabetes mellitus, dyslipidemia, hypertension, smoking, and chronic kidney disease. When the primary physicians found increased carotid intima-media thickness (cIMT) in an ultrasound examination, they were supposed to refer the patient to our institution for further examination. Since 2011, a professor of the department of hepatology, diabetes, metabolism and endocrinology in Saga University Hospital, which is another major medical center in our area, supported our screening strategy in diabetic patients, which led to an increase in the number of the patients who were referred to our institution with abnormal carotid ultrasound findings, and an increase in the number of referring primary physicians (Fig. 1). The patients who were referred to our institution were examined by the staff of our department, and underwent coronary computed tomography angiography (CCTA), unless they had any contraindications. Consequently, a total of 135 asymptomatic patients were referred to our institution with abnormal carotid ultrasound findings and underwent CCTA between January 2009 and December 2013. We retrospectively collected and analyzed their data in the present study. The exclusion criteria included a history of coronary artery disease, or a sophisticated cardiac examination, such as myocardial perfusion scintigraphy, CCTA, and coronary angiography.

### Carotid ultrasound examination

Carotid ultrasound examinations of asymptomatic patients with multiple coronary risk factors were performed at each private clinic. The primary care physicians were encouraged to refer the patients with carotid plaque, the presence of which was determined based on the presence of at least two of the following factors: a max-cIMT ≥ 1.5 mm, a change in the carotid wall surface contour, or a focal change in the echogenicity of the carotid wall [6, 7, 16], while the selection of patients and the final decision of referral to our institution was left to the discretion of the primary physicians.

### Assessment of coronary artery disease by CCTA

All patients underwent CCTA with a 64-slice scanner or a 320-slice scanner (Aquilion; Toshiba Medical Systems, Tochigi, Japan) following the institutional standard protocol, including pretreatment with β-blockers and sublingual nitrates if necessary [17]. Reconstruction was performed using the phases with the fewest motion artifacts and the images were transferred to a remote workstation for post-processing, and interpretation using a specialized software program (Ziostation; Ziosoft, Tokyo, Japan). The presence of coronary atherosclerosis and luminal narrowing in each segment was visually graded into four classes using a 15-segment model according to the American Heart Association classification: 0% (no plaque), 1-50% stenosis (mild stenosis), 51-75% stenosis (moderate stenosis), and > 75% stenosis (severe stenosis).

A recommendation for the subsequent management of each patient was communicated to their primary physician. Diagnostic coronary angiography was recommended in patients with severe stenosis, while stress cardiac perfusion scintigraphy or diagnostic coronary angiography (if possible) was recommended in patients with moderate stenosis or severe calcified lesions. For patients with mild stenosis or normal coronary arteries, no further imaging studies were recommended. A recommendation for revascularization was based on best clinical judgment of our heart team.

We retrospectively divided our patients into the significant stenosis (SIG) group and NOT group according to their coronary severity. The SIG group included patients having > 50% coronary stenosis on the coronary angiogram or on CCTA if patients did not undergo coronary angiography. All remaining patients were included in the NOT group.

### Statistical analyses

The quantitative data are presented as the mean ± SD. The qualitative data are presented as frequencies. Continuous variables were compared between the two groups using the Mann-Whitney test. Categorical variables were compared with the Chi-square test. A multivariate logistic regression analysis was developed to identify the clinical factors associated with > 50% coronary stenosis, which was classified in the SIG group, using a backward stepwise procedure. The clinical factors with a P value of < 0.10 were included in the univariate analysis and then eliminated in a stepwise manner if they did not reach statistical significance with a P value of 0.05. All probability values were two-tailed, and a value of P < 0.05 was considered to be statistically significant. All statistical analyses were performed using the SPSS software program (SPSS, Inc., Chicago, IL).

## Results

**Figure 2:**
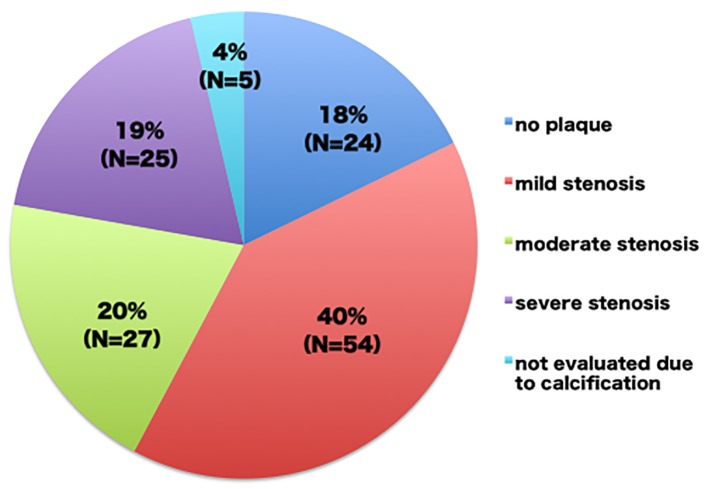
The coronary computed tomography angiography findings in the referred patients with multiple coronary risk factors and carotid atherosclerosis.

**Figure 3:**
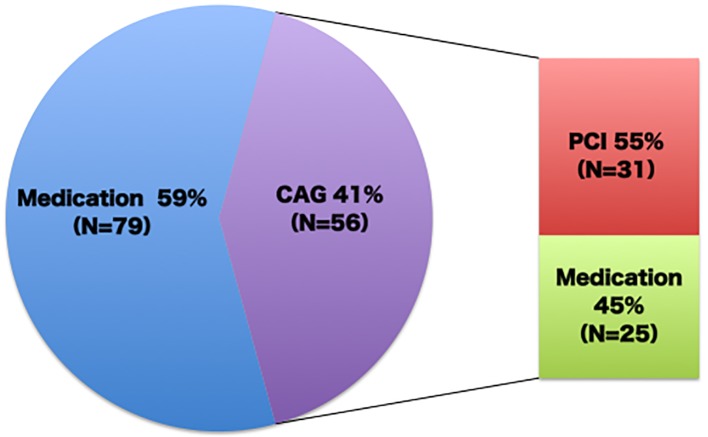
Patient management as the result of coronary artery disease screening in the referred patients with multiple coronary risk factors and carotid atherosclerosis.

The clinical characteristics of the patients are shown in Table 1. The number of coronary risk factors was 4.1 ± 1.2, but they were relatively controlled. The maximum cIMT was < 1.5 mm in 28 (21%) patients, but they had significant plaques in the carotid arteries. The CCTA findings revealed that most of the patients had coronary plaque and 19% of the patients had severe stenosis (Fig. 2). As a result, diagnostic coronary angiography was performed in 56 (41%) patients, including all patients whose stenosis was not evaluated on CCTA due to calcification. According to the results of coronary angiography, 20 (15%) patients having mild or moderate stenosis on CCTA were classified into the SIG group, while one (1%) patient having severe stenosis on CCTA was classified into the NOT group. Consequently, 31 patients underwent coronary revascularization; coronary intervention was eventually needed in for 23% of the patients, who were asymptomatic patients with abnormal cIMT values who were referred by their primary physicians (Fig. 3).

**Table 1:** Patient Characteristics

	Total (n = 135)	SIG (n = 48)	NOT (n = 87)	P value
Age (years)	69 ± 11	69 ± 9	68 ± 8	0.68
Male	102 (76)	39 (81)	63 (72)	0.25
Coronary risk factors				
Hypertension	85 (63)	32 (67)	53 (61)	0.51
Dyslipidemia	84 (62)	32 (67)	52 (60)	0.43
Diabetes mellitus	83 (62)	32 (67)	51 (59)	0.36
Insulin	15 (11)	4 (8)	11 (13)	0.45
Oral antihyperglycemic agents	56 (42)	21(44)	35 (40)	0.69
Smoking	63 (47)	25 (52)	87 (100)	0.35
Chronic kidney disease	5 (4)	1 (2)	4 (5)	0.46
Total cholesterol (mg/dL)	188 ± 37	180 ± 36	181 ± 61	0.46
LDL cholesterol (mg/dL)	108 ± 31	103 ± 32	113 ± 31	0.17
HDL cholesterol (mg/dL)	55 ± 16	51 ± 15	58 ± 16	0.01
LDL/HDL	2.12 ± 0.78	2.14 ± 0.82	1.80 ± 0.30	0.05
Systolic blood pressure (mm Hg)	138 ± 20	135 ± 20	140 ± 20	0.23
Diastolic blood pressure (mm Hg)	81 ± 12	78 ± 13	83 ± 11	0.04
Number of the risk factors	4.1 ± 1.2	4.4 ± 1.2	4.0 ± 1.2	0.13
Antihypertensive drugs	81 (60)	35 (73)	46 (53)	0.02
Number of the drugs	1.2 ± 1.2	1.4 ± 1.2	0.99 ± 1.2	0.15
Angiotensin converting enzyme inhibitor	5 (4)	1 (2)	4 (5)	0.46
Angiotensin receptor blocker	58 (43)	26 (54)	32 (37)	0.05
β blocker	16 (12)	8 (17)	8 (9)	0.20
α blocker	9 (7)	3 (6)	6 (7)	0.89
Calcium channel blocker	50 (37)	24 (50)	26 (30)	0.02
Diuretic agent	12 (9)	5 (10)	7 (8)	0.64
Aldosterone antagonist	2 (2)	0 (0)	2 (2)	0.29
Statin	50 (37)	18 (38)	32 (37)	0.93
Carotid intima-media thickness (mm)	2.00 ± 0.63	2.14 ± 0.71	1.99 ± 0.56	0.10

Data are mean ± SD or number of patients (percentage). P values indicate the SIG group vs. the NOT group. LDL: low-density lipoprotein; HDL: high-density lipoprotein. The risk factors were defined as male gender, age older than 45 years in men and 55 years in women, diabetes mellitus, dyslipidemia, hypertension, smoking, and chronic kidney disease.

The patients in the SIG group had significant lower high-density lipoprotein cholesterol levels, a higher ratio of low-density lipoprotein cholesterol levels to high-density lipoprotein cholesterol levels, and lower diastolic blood pressure levels. The prescription percentage of calcium channel blockers was significantly higher in the SIG group, and that of angiotensin receptor blockers also tended to be higher. In addition, there was a tendency of higher cIMT values in the SIG group (Table 1). Then, these clinical factors were examined to identify independent predictors for > 50% coronary stenosis in the SIG group with a multivariate logistic regression analysis. As a result, the ratio of low-density lipoprotein cholesterol levels to high-density lipoprotein cholesterol levels (odds ratio (OR): 3.46, 95% CI: 1.59 - 7.55, P < 0.01), use of calcium channel blockers (OR: 2.83, 95% CI: 1.20 - 6.70, P = 0.02) and the value of the diastolic blood pressure (OR: 0.96, 95% CI: 0.93 - 0.99, P = 0.04) were found to be related to > 50% coronary stenosis.

## Discussion

The present study revealed that the ultrasound screening of asymptomatic patients having multiple risk factors for coronary artery disease by their primary care physicians was associated with a high prevalence of coronary artery disease and the increased performance of coronary angiography and coronary revascularization. Similar results have previously been reported, but those earlier findings were limited due to the fact that they were based on the data obtained from one university hospital [11-13]. A carotid ultrasound examination is an easy, non-invasive method of obtaining images of the carotid arteries; however, the precise measurements that are described in previous studies require the training of sonographers and the maintenance of quality control. Thus, it was unclear whether the screening was useful for daily practice in the real world. Our results support that this strategy can be implemented in the clinics of primary care physicians.

Although cardiovascular risk assessment based on conventional risk factors is generally recommended for predicting cardiovascular risk, the predictive ability of this approach is only moderate [18, 19]. In contrast, imaging modalities like myocardial perfusion scintigraphy and CCTA can determine disease severity with a high degree of sensitivity and specificity; however, these modalities are not practical for the screening of all high-risk patients [20]. Therefore, a non-invasive and inexpensive screening method with a better than moderate predictive ability is required. Carotid ultrasound and the measurement of coronary artery calcification by computed tomography have been proposed as such methods. In the present study, carotid ultrasound was found to be a suitable screening tool because cIMT is reported to be a more sensitive measurement of atherosclerosis than coronary artery calcification [21] and carotid ultrasound was available at the clinics of most of the primary care physicians in our area.

A multivariate logistic regression analysis demonstrated that the ratio of low-density lipoprotein cholesterol levels to high-density lipoprotein cholesterol levels, use of calcium channel blockers and the value of the diastolic blood pressure were predictors of > 50% coronary stenosis, which was classified in the SIG group. Previous studies have reported that the ratio of low-density lipoprotein cholesterol levels to high-density lipoprotein cholesterol levels was a predictor for coronary lipid-rich plaque and also one of the independent predictors for coronary artery stenosis in asymptomatic type 2 diabetic patients [22, 23]. Thus the ratio may be a useful marker for primary care physicians to manage patients with multiple coronary risk factors. On the other hand, the use of calcium channel blockers was a significant predictor in the present study, because vasodilation might reduce myocardial ischemia and prevent angina. Similarly, the higher but relatively controlled diastolic pressure of 83 ± 11 mm Hg in the SIG group, compared to 78 ± 13 mm Hg in the NOT group, might be protective to maintain the coronary blood flow in patients with coronary artery disease.

CCTA is a non-invasive imaging modality for detecting coronary artery disease with a high negative prediction value and a good prognostic value [24-28]. It may therefore be a suitable examination for patients in whom abnormal carotid ultrasound findings are detected in coronary artery screening. On the other hand, myocardial perfusion scintigraphy is another imaging modality, which should be utilized for the screening of coronary artery disease in order to improve its predictive ability. Although myocardial scintigraphy remains apparently more applicable in patients with chronic kidney disease than CCTA, recent studies have reported the better performance of CCTA in the diagnosis and for predicting the prognosis for most patients [29, 30]. Moreover, CCTA images can provide a great deal of valuable information about coronary artery disease, which can lead to successful coronary revascularization procedures [31], thus this strategy seems to achieve the best flow from screening to treatment.

CCTA and myocardial scintigraphy are the best imaging modalities for the diagnosis of coronary artery disease, and some studies have shown their feasibility in the first stage of the screening of patients with coronary risk factors for coronary artery disease [32-34]. However, screening with myocardial scintigraphy did not achieve a decrease in cardiac death or nonfatal myocardial infarction in the DIAD study [35]. Similarly, the CCTA screening of asymptomatic patients with diabetes did not reduce the rates of all-cause mortality, non-fatal myocardial infarction, or unstable angina requiring hospitalization [36]. Actually, screening strategies that utilize imaging examinations are not practical in the real world, due to the high cost and the associated radiation exposure. In addition, the percentages of screened patients with a moderate or large defect on stress myocardial perfusion imaging and moderate or severe stenosis on CCTA were only 6.3% and 22.6%, respectively. The low prevalence of coronary artery disease in their screened patients might be a factor that could not demonstrate the superiority of screening. On the contrary, 39% patients in the present study had moderate or severe coronary stenosis, and therefore the high prevalence of coronary artery disease in the patients that was detected based on abnormal carotid ultrasound findings supported the usefulness of carotid ultrasound as the first stage in screening for coronary artery disease. We therefore need to investigate whether the present screening strategy could reduce the incidence of cardiovascular events.

Another important advantage of CCTA is the association between the severity of the findings and the increased use of preventive cardiovascular medications [37]. Although myocardial perfusion scintigraphy reveals myocardial ischemia, CCTA is able to identify coronary plaque in the absence of myocardial ischemia. The identification of coronary plaque will lead to the prescription of preventive cardiovascular medications, even for patients without severe stenosis [36], and the visualization of coronary atherosclerosis may motivate patients to make lifestyle modifications, and result in improved prognosis and the reduction of medical care costs.

The present study is associated with some important limitations. First, it was a retrospective study that was performed at a single institution, and it involved a limited number of primary physicians. Second, the carotid ultrasound examinations were only reported at private clinics, and the measurements were not confirmed at our institution. Third, because the selection of patients for the carotid ultrasound examinations was completely left to the discretion of the primary care physicians, the present study may be associated with some deviation and selection bias. However, the process of this study reflected real patient care in our area. Fourth, all the coronary lesions on CCTA were not confirmed by coronary angiography, and the evaluation of coronary ischemia was not mandatory for the coronary intervention; however, the discussion with our heart team was mandatory when determining the treatment strategy. Finally, we did not set a control group for the coronary artery disease screening, and we did not follow up patients with negative carotid ultrasound findings. Therefore, we need to demonstrate that the present screening strategy is capable of improving the prediction of coronary artery disease, and reducing the incidence of cardiovascular events and medical costs in comparison to conventional risk assessment. However, the high prevalence of coronary artery disease and the increased probabilities that the patients of the present study undergoing coronary angiography and revascularization seem to indicate the practicality of screening for coronary artery disease by primary care physicians.

### Conclusions

The use of carotid ultrasound examination by primary care physicians is a useful and feasible strategy for coronary artery disease screening for asymptomatic patients with multiple coronary risk factors.
